# Extracellular chaperone networks and the export of J-domain proteins

**DOI:** 10.1016/j.jbc.2022.102840

**Published:** 2022-12-26

**Authors:** Janice E.A. Braun

**Affiliations:** Department of Biochemistry and Molecular Biology, Hotchkiss Brain Institute, University of Calgary, Calgary, Alberta, Canada

**Keywords:** JDP, DnaJ, J-domain proteins, molecular chaperones, EVs, extracellular chaperones, extracellular vesicles, exosomes, misfolded proteins, CNS, central nervous system, ER, endoplasmic reticulum, EV, extracellular vesicle, HEK293T, human embryonic kidney 293T cell line, Hsp70, heat shock protein 70, JDP, J-domain protein, sHSP, small heat shock protein

## Abstract

An extracellular network of molecular chaperones protects a diverse array of proteins that reside in or pass through extracellular spaces. Proteins in the extracellular milieu face numerous challenges that can lead to protein misfolding and aggregation. As a checkpoint for proteins that move between cells, extracellular chaperone networks are of growing clinical relevance. J-domain proteins (JDPs) are ubiquitous molecular chaperones that are known for their essential roles in a wide array of fundamental cellular processes through their regulation of heat shock protein 70s. As the largest molecular chaperone family, JDPs have long been recognized for their diverse functions within cells. Some JDPs are elegantly selective for their “client proteins,” some do not discriminate among substrates and others act cooperatively on the same target. The realization that JDPs are exported through both classical and unconventional secretory pathways has fueled investigation into the roles that JDPs play in protein quality control and intercellular communication. The proposed functions of exported JDPs are diverse. Studies suggest that export of DnaJB11 enhances extracellular proteostasis, that intercellular movement of DnaJB1 or DnaJB6 enhances the proteostasis capacity in recipient cells, whereas the import of DnaJB8 increases resistance to chemotherapy in recipient cancer cells. In addition, the export of DnaJC5 and concurrent DnaJC5-dependent ejection of dysfunctional and aggregation-prone proteins are implicated in the prevention of neurodegeneration. This review provides a brief overview of the current understanding of the extracellular chaperone networks and outlines the first wave of studies describing the cellular export of JDPs.

A network of molecular chaperones ensures protein homeostasis (proteostasis) by mediating the folding, trafficking, sequestration, and turnover of cellular proteins. In the human genome, over 300 genes encode for molecular chaperones, which are classified into chaperone families based on structure (1, 2). The major chaperone families are HSP110 (HSPH), Hsp90 (HSPC), heat shock protein 70 (Hsp70) (HSPA), Hsp60, TRiC (CCT), small heat shock proteins (sHSPs) (HSPB), and J-domain proteins (JDPs) with numerous chaperones falling outside these main categories. The JDP molecular chaperone family, also called the DnaJ protein family and the J protein family, is the largest and most versatile chaperone family. Each JDP contains a J-domain that tethers Hsp70 with client proteins and enhances the ATPase activity of Hsp70. In addition, JDPs contain distinct subdomains that mediate interactions with a broad range of proteins. Hsp70s are a ubiquitous and abundant family of molecular chaperones implicated in diverse transactions with substrate proteins. By far, the largest number of JDP activities are centered around proteostasis, although some JDPs have evolved to specialize in the import of proteins into the endoplasmic reticulum (ER) and mitochondria (3, 4), gene splicing (5) or mRNA processing (6). The multifunctionality of JDPs is an established feature of molecular chaperone networks and has been discussed in detail (7, 8, 9, 10, 11, 12, 13). This review provides a brief overview of extracellular chaperone subnetworks. Recent studies investigating JDP export are discussed, and the emerging questions regarding the activities of extracellular JDPs are highlighted.

## Secretion of chaperones

Roughly, a dozen unrelated chaperones make up the extracellular subnetwork that controls proteins residing in, or passing through extracellular spaces, and the list is growing (Table 1). Extracellular proteins originate from multiple sources and collectively have a high degree of complexity and diversity. Gene mutations or errors in transcription and translation can result in the secretion of damaged and misfolded proteins, although all proteins are susceptible to misfolding in the extracellular milieu. Misfolded proteins are frequently toxic and often possess an inherent capacity to aggregate (14, 15). Safeguarding cells from toxic proteins and controlling extracellular protein aggregation is extremely important. In extracellular spaces, chaperones scavenge for and sequester misfolded proteins, thereby eliminating toxicity and preventing the formation of aggregates. When extracellular chaperones are overwhelmed, the activities of proteins with unstructured and unstable conformations may dominate, manifesting in disease (16). For the most part, extracellular proteostasis has been examined through the lens of individual chaperone function; however in actuality, it is the collaborative work of a diverse collection of chaperones that maintains the integrity of proteostasis. Local fluctuations (*e.g.*, transient upregulation) in the expression of subsets of extracellular chaperones within the various extracellular spaces are widely recognized, and the secretion of several chaperones has been nicely summarized in the literature, that is, clusterin (apolipoprotein J) (17, 18), haptoglobulin (16), α_2_-macroglobulin (19), transthyretin (20), 7B2 (21, 22), proSAAS (22), progranulin (22, 23, 24), neuroserpin (25), apolipoprotein E (20), caseins (16), sHSPs (HspBs) (26, 27) as well as BRICHOS domain-containing chaperones (28, 29). The reader is directed to these comprehensive reviews for additional information.

**Table 1:** Examples of extracellular chaperones and their involvement in human disease

Chaperone	Colocalization with deposits	Mutations and disease
JDPs		>100 mutations (11, 13)
DnaJA1		
DnaJA2	Tau-surround aggregates (118)	
DnaJA4		
DnaJB1		Fibrolamellar hepatocellular carcinoma (119)
DnaJB2	Aβ (120)	Charcot–Marie–Tooth disease type 2 (121, 122) Distal hereditary motor neuropathy (123, 124, 125) Spinal muscular atrophy/juvenile Parkinsonism (121) Parkinson’s disease (126)
DnaJB6		Limb girdle muscular dystrophy type 1D (127, 128, 129, 130, 131, 132) Frontotemporal dementia with limb girdle muscular dystrophy (133)
DnaJB8		
DnaJB11		Polycystic kidney disease (134)
DnaJC5		Adult-onset neuronal ceroid lipofuscinosis-CLN4 (102, 103, 104, 106)
Clusterin (apolipoprotein J)	Aβ (135, 136, 137, 138, 139, 140, 141) tau (142) α-synuclein (143) TAR DNA-binding protein 43 (TDP-43) (144) Prions (145, 146) γ-tubulin (147) Drusen (148)	Alzheimer’s disease (149, 150, 151, 152, 153, 154, 155, 156, 157, 158, 159) Macular degeneration (149, 150) Parkinson’s disease (160, 161)
Apo E (apolipoprotein E)	Aβ (162)	Alzheimer’s disease-ApoE4 (152, 162, 163, 164, 165)
Haptoglobin	Aβ (166)	Parkinson’s disease (167)
α_2_-macroglobulin	Aβ (16) Prions (16)	Alzheimer’s disease (168, 169)
Transthyretin		>100 mutations (20, 170, 171)
		Familial amyloid cardiomyopathy (172) Familial amyloid polyneuropathy (173) Oculoleptomeningeal amyloidosis (174)
BRICHOS-domain chaperones		>300 proteins (29)
Bri2 Bri3 ProSP-C	Aβ (175, 176, 177)	Familial British dementia-Bri2 (178, 179) Familial Danish dementia-Bri2 (180) ITM2B-related retinal dystrophy-Bri2 (181) Frontotemporal dementia/amyotrophic lateral sclerosis-Bri2 (182) Familial interstitial lung disease-ProSP-C (183)
7B2	Aβ (184) α-synuclein (184)	
ProSAAS	Aβ (185) Tau (186, 187) α-synuclein (188)	
Progranulin		>70 mutations (23, 54)
	Aβ (189, 190)	Frontotemporal dementia (191, 192) Adult-onset neuronal ceroid lipofuscinous-CLN11 (193, 194, 195) Alzheimer’s disease (193, 196) Parkinson’s disease (193, 197) Limbic-predominant age-related TDP-43 encephalopathy (198) Gaucher disease (199) Complicated spastic paraplegia (200) Amyotrophic lateral sclerosis (201)
Neuroserpin	Aβ (25, 202))	Familial encephalopathy with neuroserpin inclusion bodies (203, 204)
sHSPs		>100 mutations (26, 27, 205, 206)
HspB1 HspB5 HspB6	Aβ (207) Tau (208) α-synuclein (209) Superoxide dismutase-1 (210) Rosenthal fibers (211))	Cataract (212, 213) Charcot–Marie–Tooth disease type 2 (214) Distal hereditary motor neuropathy type II (214, 215) Desmin-related myopathy (216) Amyotrophic lateral sclerosis (217, 218, 219) Hereditary spastic paraparesis (217) Dilated cardiomyopathy (220)

Despite the importance of extracellular proteostasis, we still have much to learn regarding the genetic, epigenetic, and molecular mechanisms that regulate extracellular chaperone expression and secretion (16, 30). Chaperones destined for export arise from a multitude of cell types, and their expression and secretion are controlled by a myriad of processes. Major sources include liver (haptoglobin, α_2_-macroglobulin, apolipoprotein E, clusterin, and transthyretin), myeloid cells (progranulin), lung (proSP-C), breast (casein), neuroendocrine cells (7B2, ProSAAS), and brain (clusterin, neuroserpin, transthyretin, Bri3, and progranulin). In addition, extracellular chaperone levels are substantially influenced by the heat shock response, the unfolded protein response, the acute phase response, as well as lactogenic hormones, exercise, and age (30). One of the most prominent extracellular chaperones, clusterin, a heavily glycosylated chaperone composed of α and β subunits linked by five disulfide bridges, is regulated by hormones, growth factors, and cytokines and is upregulated in several pathologies, including many types of cancer and neurodegenerative diseases. Micro-RNAs, DNA methylation, histone deacetylation, and multiple transcription factors including the heat shock response regulate clusterin expression (31). The sHSPs are central extracellular chaperones. In humans, there are 10 sHSPs that are differentially regulated in a tissue-specific manner and three, HspB1 (Hsp27), HspB5 (αB-crystallin), and HspB6 (Hsp20), are prominent extracellular chaperones (27). Some sHSPs, for example HspB1, are upregulated by the heat shock response. Secretion of HspB1 is also upregulated in several pathologies, including atherosclerosis, multiple sclerosis, Parkinson’s disease, and diabetes. Extracellular HspB5 levels are higher in multiple sclerosis, and extracellular HspB6 levels are upregulated in cardiomyopathy (27). Each sHSP contains an evolutionarily conserved core α-crystallin domain characterized by antiparallel β-sheets that is flanked by variable N-terminal and C-terminal regions; these features underlie the inherent structural plasticity of sHSPs. Functionally, individual sHSPs operate as either homomeric or heteromeric complexes (26, 27, 32, 33). Although the expression of the individual members of the extracellular chaperone network is incompletely characterized, it is apparent that the expression of extracellular chaperones is subject to tight control.

**Figure 1:**
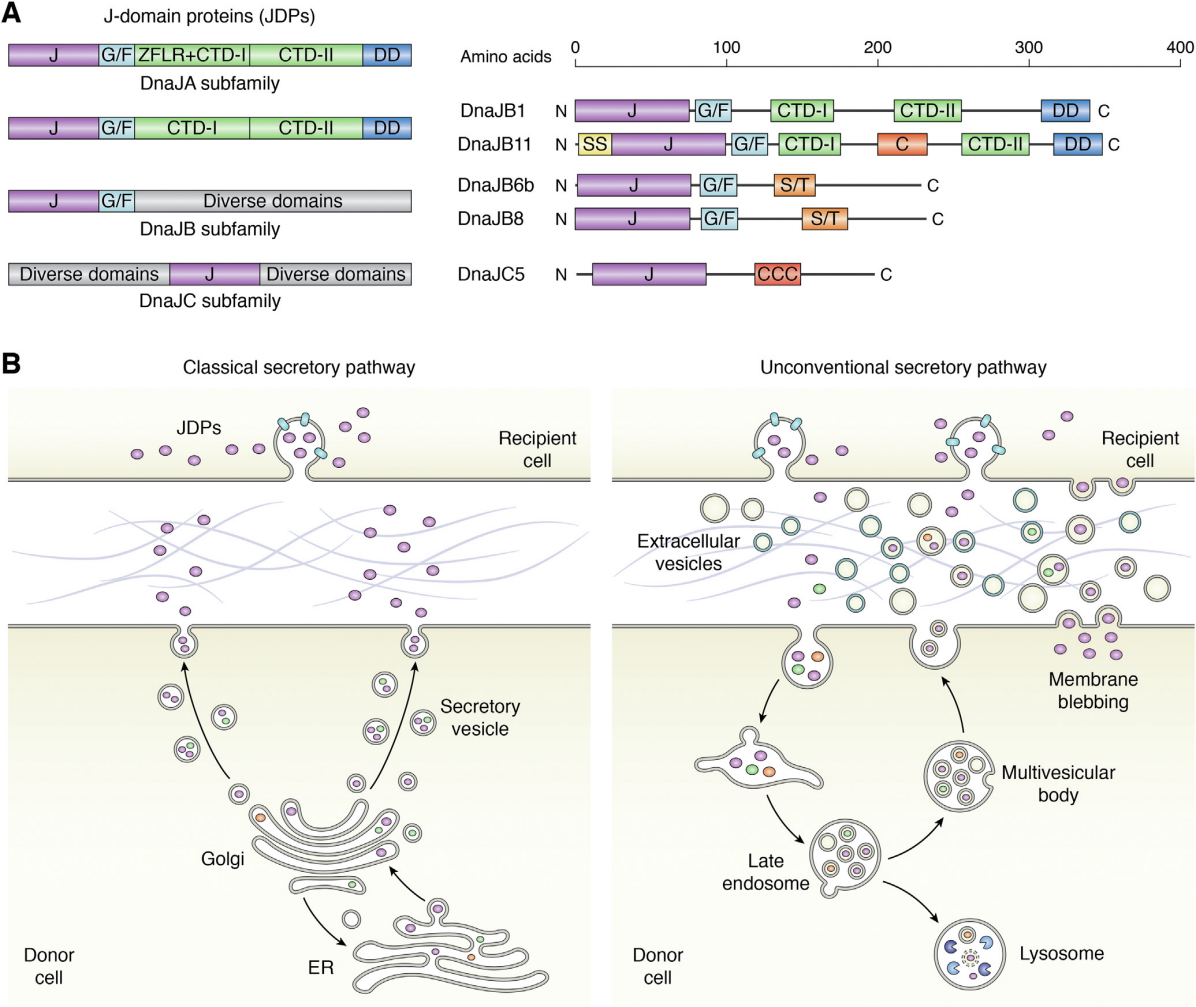
**Secretion of JDPs.***A*, schematic *cartoon* of the structural similarities and differences between the subclasses of JDPs DnaJA, DnaJB, and DnaJC. Exported JDPs are indicated. The defining domain architecture of the subclasses is shown. The DnaJCs have the greatest diversity in size and architecture, as the J domain may be present anywhere in the structure of the protein. Adapted from Ref. (8). *B*, schematic overview of the secretory pathways for JDPs in mammalian cells. A diverse array of proteins and molecular chaperones transverse the extracellular space. In the classical secretory pathway, JDPs containing signal sequences are translocated into the endoplasmic reticulum, pass through the Golgi complex, and are released from the plasma membrane. In the unconventional secretory pathway, JDPs are exported in extracellular vesicles (EVs). EVs that are heterogeneous in both size and content are produced through the endosomal pathway by fusion of multivesicular bodies (MVBs) with the plasma membrane (exosomes) as well as by the outward blebbing and pinching of the plasma membrane. Following secretion in the extracellular space, EV and nonvesicular JDPs are taken up by recipient cells through multiple mechanisms. C, cysteine-rich region; CCC, cysteine string region; CTD-I and CTD-II, C-terminal domains involved in substrate binding; DD, dimerization motif; G/F, glycine/phenylalanine linker; J, J-domain; JDP, J-domain protein; SS, signal sequence; S/T, serine–threonine–rich region; ZFLR, zinc finger–like region.

Chaperones and their client proteins enter the extracellular space through the classic secretory pathway, the unconventional secretory pathways, as well as cell lysis (Fig. 1). Classic secretion of chaperones is initiated by signal peptide–dependent protein translocation into the lumen of the ER, followed by passage through the Golgi complex and release from the cell surface. Differences exist in how chaperones travel through the secretory pathway. Clusterin and apolipoprotein B are constitutively secreted, whereas 7B2 and proSAAS are packaged into regulated secretory granules and released following stimulation (17, 18, 20, 21, 22, 34). The BRICHOS domain chaperones, Bri2 and ProSP-C (surfactant protein C), are resident ER transmembrane proteins that move through the secretory pathway following proteolytic cleavage of a transmembrane linker (28, 29). In the absence of a classic secretory signal sequence, sHSPs, Hsp70, and Hsp90 are either secreted in extracellular vesicles (EVs) or are actively translocated across the plasma membrane (26, 27, 35, 36, 37, 38). EVs are membrane vesicles released from most types of cells. They transfer a diverse and tightly packaged array of proteins, lipids, and RNA over both short and long distances (39, 40). In the central nervous system (CNS), EVs have been implicated in synaptic plasticity, neurodevelopment, myelination, as well as neural maintenance by shuttling cellular materials between brain endothelium, astrocytes, microglia, and neurons (19, 41, 42, 43, 44, 45, 46, 47, 48, 49, 50). In this manner, EVs are involved in the cell-to-cell delivery of biologically active compounds, along with the removal of cellular waste (38, 51). That is, EVs are capable of transporting both “care packages” as well as “toxic cellular trash” between cells. To date, many experimental procedures have resulted in the coisolation of EV subpopulations that arise from different intracellular compartments and sometimes different cell types, which has limited our understanding of the function of specific EV subpopulations (38, 51). Several types of EVs have been described, including exosomes, microvesicles, exophers, large oncosomes, and apoptotic bodies (38, 40), and it remains to be determined how chaperones are distributed in these EVs. Operationally, some subpopulations of EVs bud from the plasma membrane, whereas other EVs arise from membrane internalized into endosomes to form multivesicular bodies that fuse at the cell surface to secrete small intraluminal vesicles (Fig. 1).

The chaperone progranulin is secreted through the classical secretory pathway (52) as well as EVs (53). Structurally, progranulin is comprised of seven and a half granulin domains arranged as “beads-on-a-string” that are cleaved into individual ∼6 kDa granulins by extracellular or lysosomal proteases. Functionally, extracellular progranulin is taken up by many cell types and trafficked to lysosomes, where it chaperones cathepsin D, β-glucocerebrosidase, and prosaposin (22, 23). Progranulin and the cleaved granulins are both biologically active, although with independent actions; for example, progranulin is reported to have anti-inflammatory roles, whereas granulins have proinflammatory functions (23, 54).

Once released, exported chaperones enter an extracellular space of low ATP availability and fluctuating pH, where sheer stress, oxidative stress, and pathogen invasion can represent proteostasis challenges (16). At present, there is no evidence to substantiate active protein refolding in the extracellular space. Rather, it is speculated that extracellular chaperones act as “holdases,” meaning that they prevent the aggregation and precipitation of proteins, but are not able to refold misfolded proteins (22). Once present in the extracellular space, chaperones “patrol” for partially unfolded proteins with exposed hydrophobic regions. Structurally, the exposed hydrophobicity of misfolded proteins is an initiating factor for the formation of protein aggregates and deposits. By forming complexes with aggregation-prone proteins, extracellular chaperones likely prevent partially unfolded proteins from aggregating and assist in their internalization in receptive cells, an activity that may be modified by the glycosylation and lipidation status of the chaperones. Once internalized, the chaperone–client complexes either undergo structural refolding or are degraded by the ubiquitin–proteasome and autophagy–lysosome pathways (16, 22). Haptoglobin is best known as an extracellular chaperone that binds free hemoglobin released from erythrocytes and facilitates its removal from the blood (16). Transthyretin targets thyroxin and the retinol-binding protein complex (20). 7B2 and proSAAS act on the prohormone convertases, PC2 and PC1/3, respectively, which, in turn, regulate maturation of neuroendocrine peptides (21, 22). While these chaperones may have initially coevolved with specialized clients, in many cases, their antiaggregating actions are now known to extend beyond the primary client protein (16). On the other hand, clusterin and sHSPs have broad client protein–binding profiles, and α_2_-macroglobulin is a broad-spectrum chaperone that rapidly inhibits and clears extracellular proteases from the circulation (16, 17, 26, 27, 34). The broad and target protein-binding profiles of extracellular chaperones suggest that these chaperones utilize overlapping mechanisms to maintain proteostasis.

The biological importance of extracellular chaperones is reinforced by findings that several diseases are caused by mutations in these proteins (Table 1). Ineffective chaperones or an excess of misfolded extracellular proteins can lead to deposits of protein aggregates (14, 15). Protein aggregates are a hallmark feature of many human diseases and extracellular chaperones, such as clusterin and sHSPs, often found sequestered in protein deposits, perhaps because of a failed attempt to keep the aggregating proteins soluble (Table 1). A large number of studies has demonstrated overwhelmingly that extracellular chaperones regulate protein aggregation *in vitro* (for review, see Refs. (16, 18, 22, 23, 25, 26, 28, 29, 55)). In parallel, studies have shown that toxic protein aggregates propagate between contiguous neuroanatomical regions in prion disease, Alzheimer’s disease, Parkinson’s disease, amyotrophic lateral sclerosis, Huntington’s disease, traumatic brain injury, and stroke. Infectious prions are transmissible pathogens that cause Creutzfeldt–Jakob disease in humans, chronic wasting disease in elk and mule deer, scrapie in sheep and bovine spongiform encephalopathy in cows, and EVs are proposed to mediate the spread of infectious prions in the CNS (48, 49, 56, 57, 58). Other studies report that EV transmission mediates the “prion-like spread” of noninfectious and misfolded disease-causing proteins, for example, amyloid precursor protein and Aβ (59, 60, 61, 62), tau (63, 64, 65), superoxide dismutase-1 (66, 67), TAR DNA-binding protein 43 (68), α-synuclein (69, 70) and Huntingtin (71, 72). Little is known about how EVs and concomitant nonvesicular protein aggregates spread pathology in the CNS, and further research is needed to understand how extracellular chaperones influence the propagation of misfolded proteins *via* EVs or other pathways.

## JDPs

The picture emerging is that the extracellular chaperone subnetwork serves as a checkpoint for the movement of a diverse array of proteins between cells, and if compromised, dysfunction may ensue. JDPs have recently joined the list of chaperones that are exported through classical (73) and unconventional (71, 72, 74, 75, 76, 77, 78) secretory pathways. In addition to these studies, 24 JDPs and an additional eight mRNAs encoding JDPs are reported to be present as EV cargo in the Vesiclepedia and Exocarta databases, suggesting that the extent of JDP export has not been fully appreciated (Vesiclepedia database: http://microvesicles.org; Exocarta database: http://www.exocarta.org) (79).

The evolutionarily conserved JDP family is found in yeast, bacteria, plants, mammals, and viruses and exhibits wide-ranging functional versatility (8, 9). Over 1500 unique domains have been identified within JDPs in all kingdoms, and interactions with diverse proteins are facilitated by these different domains (13). While substrates of some JDPs are well characterized, others remain unknown. The human genome encodes 50 JDPs that are mostly constitutively expressed, whereas some are regulated by cell stress (1, 9). One domain, the signature J domain, is found in all members of the JDP family and facilitates the binding and activation of Hsp70 ATPases, a family of conserved ubiquitously expressed chaperones. Structurally, the J domain is comprised of four alpha helices and is approximately 70 amino acids in length. Within the J domain, a conserved histidine, proline, aspartate motif located between helices II and III interacts with Hsp70s, initiating intramolecular activation of the ATPase enzymatic activity (8). There is no functional one-to-one correspondence between Hsp70s and JDPs. In humans, there are 13 Hsp70s that are expressed in different cellular compartments that have multiple JDP partners (1).

Intracellularly, Hsp70s operate *via* iterative cycles of client protein binding and release that are tied to diverse functional outputs (80). When Hsp70s are ATP bound, protein substrates bind and release rapidly; however, when Hsp70s are ADP bound, client proteins remain tightly bound. Hsp70 association with and release from substrate proteins promote folding, although many functional questions remain. The association and dissociation of Hsp70s and client proteins are coupled to ATP hydrolysis, and although the ATPase activity of Hsp70s is inherently slow, it is allosterically stimulated by JDPs (7, 8). The energy released from ATP hydrolysis drives client protein binding and release cycles. This occurs as the histidine, proline, aspartate motif within the J domain of a client-carrying JDP interacts with ATP-bound Hsp70, which stimulates the hydrolysis of Hsp70-bound ATP. In parallel, the client protein is transferred from the JDP to Hsp70, and Hsp70 is converted from an ATP-bound state to an ADP-bound state, which stabilizes binding of the protein substrate. Next, nucleotide exchange factors enable ADP release, client proteins dissociate when Hsp70 shifts from the ADP-bound to ATP-bound state, and Hsp70 is reset for another cycle of client protein binding and release. ATP levels are several orders of magnitude lower in extracellular fluids compared with the cell interior (16), and these low levels of ATP suggest that JDPs promoting proteostasis in the extracellular space may be less dependent upon the JDP-Hsp70 cycle of client protein binding and release.

Three JDP classes, DnaJAs, DnaJBs, and DnaJCs, together drive the diverse cellular functions of Hsp70s (Fig. 1) (7). There is high structural similarity among human DnaJAs, which are closely related to prokaryotic *Escherichia coli* DnaJ (8). DnaJAs have an N-terminal J domain, a glycine/phenylalanine region and two C-terminal β barrel domains that contain a client-binding domain, a dimerization domain, and a zinc finger motif. DnaJBs are similar in architecture, often having client binding and dimerization domains but lack the zinc finger domain. The DnaJCs have the greatest diversity in size and architecture, possessing domains and motifs not found in the DnaJA and DnaJB classes, and with the J domain placed anywhere in the structure of the protein. In humans, DnaJC is the class with the largest number of members. Evidence suggests DnaJCs are functionally distinct and cannot be replaced by other JDPs, although, in many cases, information regarding JDP specificity is lacking. It is also becoming clear that different JDPs may target the same protein substrate, thereby working cooperatively for conformational purposes. The heterogeneity in architecture, localization, and expression levels of JDPs is responsible for maintaining diverse cellular functions (8, 9). Not surprisingly, changes in JDP expression and mutations in JDPs cause disease. In humans, altered JDP expression is found in cancer cells (13), mutations in distinct JDP genes manifest as dissimilar diseases (10, 13) and elevated levels of autoantibodies against JDPs are reported in sera of patients with ulcerative colitis and atherosclerotic disease (81, 82).

## DnaJA export

Data acquired in different laboratories and different cell models demonstrate that DnaJAs are exported in EVs. Proteomic analysis reveals that DnaJA1, DnaJA2, and DnaJA4 are present in small EVs released from mouse brain (47). DnaJA1 and DnaJA2 are present in small EVs from rat hippocampal neurons (45) and glioblastoma Gli36 cells (83). Western blot analysis demonstrates that small EVs from Neuro2A cells contain DnaJA1 and DnaJA2 (74). Both small- and medium-sized EVs from human primary monocyte–derived dendritic cells equally contain DnaJA1 and DnaJA2, and small and large EVs from human colon DKO-1 cells equally contain DnaJA2 (84). The Vesiclepedia database currently lists >370 entries identifying EVs containing DnaJAs or mRNA-encoding DnaJAs, suggesting that DnaJAs regularly transit between cells. Overall, DnaJAs are most often reported in EVs from cancer cells, such as breast, ovarian, skin, colorectal, bladder, kidney, lung, prostrate, and brain.

## DnaJB1 export

DnaJB1 targets a broad range of client proteins. Of all the JDPs, the intracellular activities of DnaJB1 are the most extensively studied, and it is a key chaperone of the heat shock response. More than 80 entries identifying EVs containing DnaJB1 or mRNA-encoding DnaJB1 are listed in the Vesiclepedia database. For example, Gli36 glioblastoma cells release DnaJB1 in small EVs, whereas DKO-1 colon cells release DnaJB1 in both small and large EVs (83). Interestingly, brain endothelial cells release DnaJB1 in medium EVs, and although tumor necrosis factor treatment does not increase DnaJB1 export from endothelial cells, it does increase the EV export of other JDPs, such as DnaJA2 and DnaJC13 (46). Work by Nagai *et al.* (74) reveals that when the heat shock response is activated, DnaJB1 is exported in small EVs from Neuro2A, SH-SY5Y, U373MG, and C6 cells and that the J domain of DnaJB1 is required for export. While Hsp70, Hsp90, DnaJA1, DnaJA2, and DnaJB6a are coexported, it is not known which, if any, chaperones are copackaged with DnaJB1 (38, 84, 85). To address the role of exported DnaJB1, EVs from donor cells transfected with DnaJB1 were applied to recipient cells expressing aggregation-prone proteins, and protein aggregation was assessed over time (74). These experiments demonstrate that EV delivery of DnaJB1 decreases protein aggregation in recipient cells, suggesting activation of the heat shock response in donor cells can effectively enhance the proteostasis capacity of cells accepting DnaJB1-containing EVs.

## DnaJB2, DnaJB6, and DnaJB8 export

DnaJB2, DnaJB6, and DnaJB8 are closely related JDPs that are not significantly activated by the heat shock response. DnaJB8, a testis-enriched JDP, is found in small EVs from colon cancer cells resistant to the anticancer agent, oxaliplatin. Work by Zhang *et al*. (76) shows that the delivery of DnaJB8-containing EVs conveys oxaliplatin resistance to recipient cells. To date, the Vesiclepedia database has 68 and 23 entries reporting the presence of DnaJB6 and DnaJB2 in EVs, respectively. In cellular and mouse models of Huntington’s disease, DnaJB2 and DnaJB6 reduce mutant Huntingtin aggregation and delay the onset of Huntington’s disease, whereas other JDPs do not significantly suppress aggregation (86, 87, 88, 89, 90). Work by Zuhorn *et al.* reveals that mouse neural stem cells export DnaJB6b, an alternatively spliced DnaJB6 isoform, in small EVs. To address the function of exported DnaJB6b, EVs from donor cells were injected intrathecally into the R6/2 mouse model of Huntington’s disease or applied to human embryonic kidney 293T (HEK293T) cells expressing that polyglutamine expanded Huntingtin and Huntingtin aggregation evaluated (75). These experiments demonstrate that DnaJB6 suppresses Huntingtin aggregation in cells and reduces Huntingtin inclusions in the striatum by 30%, providing proof of concept that EV delivery of DnaJB6b delays disease (75). In humans, DnaJB2 mutations are associated with several diseases, including Charcot–Marie–Tooth disease type 2, distal hereditary motor neuropathy, spinal muscular atrophy/juvenile parkinsonism, and Parkinson’s disease, whereas DnaJB6 mutations are associated with limb girdle muscular dystrophy and frontotemporal dementia (Table 1). Further research is needed to understand how JDP transit is influenced in these diseases or diseases caused by protein aggregates, such as Huntington’s disease.

## DnaJB11 export

DnaJB11/ERdj3 is one of the ER-localized JDPs involved in the folding of nascent proteins (91, 92). It is a key component of the unfolded protein response, a cellular program that increases ER quality control to prevent the secretion of dysfunctional and aggregation-prone proteins. Proteomic studies reveal nonvesicular release of DnaJB11/ERdj3 from Gli36 glioblastoma cells, but not DKO-1 colon cells, even though it is expressed in both cells (83). Work by Wiseman *et al.* demonstrates that when the unfolded protein response is activated in HEK293T, Huh7, HeLa, HepG2, CHO, and SH-SY5Y cells, a significant proportion of DnaJB11/ERdj3 transits through the secretory pathway and into the extracellular space. They show that secretion of DnaJB11/ERdj3 occurs exclusively upon activation of the activating transcription factor 6 arm of the unfolded protein response, and secretion occurs both independently or in complex with misfolded proteins (73). That is, DnaJB11/ERdj3 pre-emptively promotes extracellular proteostasis by shepherding misfolded client proteins in the ER, such as transthyretin, through the cellular secretory pathway and into the extracellular space. When secreted in the absence of a misfolded protein, DnaJB11/ERdj3 directly enhances extracellular proteostasis by binding extracellular misfolded proteins and reducing their toxicity. In mice, hepatic ER stress correlates with an elevation of DnaJB11/ERdj3 levels in the serum. These observations suggest that upregulation and secretion of DnaJB11/ERdj3 alleviates the cellular toxicity associated with a build-up of aggregation-prone proteins that are released from the cell and from aggregates in the extracellular milieu. In humans, urinary DnaJB11 is associated with glomerular ER stress, and mutations in DnaJB11 lead to polycystic kidney disease (Table 1), supporting a role for secreted DnaJB11 in opposing damage linked to extracellular protein aggregation.

## DnaJC5 export

Cysteine string protein (DnaJC5/CSPα) is a constitutively expressed and abundant presynaptic JDP that is also found in most secretory tissues (93). It is one of the most heavily palmitoylated proteins known and is essential for the maintenance of functional neurons (94, 95, 96, 97). In the absence of DnaJC5/CSPα, mice, *drosophila*, and *Caenorhabditis elegans* undergo rapid neurodegeneration (94, 95, 96). Degeneration is activity dependent, and the synapses that fire most frequently are lost first (98, 99). DnaJC5/CSPα is secreted from mouse brain (71), CAD cells (71) and HEK293T cells (77) and proteomic studies report that DnaJC5/CSPα is present in small EVs from rat hippocampal neurons (45) and both small and medium EVs from human primary monocyte–derived dendritic cells (84). The Vesiclepedia database lists >80 entries identifying EVs containing DnaJC5/CSPα or mRNA-encoding DnaJC5/CSPα. Interestingly, there are three mammalian DnaJC5/CSPα paralogs, CSPα, CSPβ, and CSPγ (95). Compared with CSPα, less is known about CSPβ, which is expressed in auditory hair cell neurons and testes or CSPγ, which is found in testes.

DnaJC5/CSPα rids cells of different disease-associated proteins by exporting them (71, 72, 77, 100, 101). Dickey *et al.* (100) have shown that DnaJC5/CSPα expression in HEK293 cells facilitates the release of wildtype and mutant forms of TAR DNA-binding protein 4, α-synuclein, and tau, and that organotypic brain slices prepared from mice lacking DnaJC5/CSPα releases less tau protein compared with slices from wildtype mice. In our own studies, we reported that DnaJC5/CSPα efficiently packages misfolded Huntingtin and superoxide dismutase-1 into EVs for secretion (71). In subsequent work, we showed that DnaJC5/CSPα exports aggregated Huntingtin protein in two distinct subtypes of EVs, sized at 180 to 240 nm and 10 to 30 μm (72). Multiple Huntingtin aggregates were readily visible in the large EVs (72). Ye *et al.* found that DnaJC5/CSPα facilitates the secretion of tau and α-synuclein from HEK293T and COS7 cells, further underscoring the versatility of DnaJC5/CSPα-mediated export (77, 78). Secretion of misfolded proteins *via* DnaJC5/CSPα is Hsp70 dependent (71, 72, 100) and interestingly, nonvesicular DnaJC5/CSPα-dependent export of misfolded proteins is also reported (78), suggesting that multiple export pathways may be involved. Given its role in neural maintenance and export of disease-causing proteins, it is feasible that DnaJC5/CSPα-mediated protein export facilitates the survival of neurons by removing accumulated toxic proteins that are not eliminated by the ubiquitin/proteasome and autophagy-lysosome pathways.

In humans, the L115R mutation or deletion of L116 in DnaJC5/CSPα lead to the lysosomal storage disease and adult-onset neuronal ceroid lipofusinosis (102, 103, 104). These mutations are located in a central cysteine string region of DnaJC5/CSPα and alter its intracellular localization (105). Duplication of a segment of the cysteine-string region also causes adult-onset neuronal ceroid lipofusinosis (106). Similar to wildtype DnaJC5/CSPα, CSPα_L115R_ and CSPα_Δ116_ are secreted, and when coexpressed with mutant Huntingtin, effectively facilitate the packaging and export of Huntingtin aggregates in EVs (71, 72). These observations were generated in cell lines, and to date, little is known about how wildtype DnaJC5/CSPα and DnaJC5/CSPα mutants facilitate the movement of pathogenic proteins in the brain. It is tempting to speculate that a toxic protein disposal pathway that becomes unregulated could lead to the unintended propagation and spread of toxic proteins. Pathways that mitigate disease by routine clearance of misfolded proteins have not yet been distinguished operationally from pathways that promote disease progression.

## Parasite and viral JDPs

To establish infection in the host, some parasites deliver parasite-encoded JDPs to the host cell, where they are thought to activate Hsp70s. For example, the parasite *Plasmodium falciparum*, which causes a severe form of malaria in humans, encodes 49 JDPs, of which 19 are predicted to be delivered to host cells and play an important role in parasite pathogenicity (107, 108, 109). Similarly, *Leishmania donovani*, a human blood parasite, delivers a number of proteins, mostly through the unconventional secretory pathway, to host cells, including JDPs (110). There are 72 JDP-coding genes in the *Leishmania* genome, and it appears that this high number of JDP-encoding genes helps the organism navigate the environmental challenges that it faces during its transmission from sand flies to warm-blooded hosts (111). From a therapeutic perspective, the large chaperone networks operating in these organisms likely represent a significant challenge to the development of antiparasitic agents. Although, the role of specific JDPs in the *Leishmania* and *Plasmodium* life cycles have not been worked out in detail, parasitic JDPs represent a demonstrable proof in principle of the biological significance of JDP export and delivery.

Of additional interest, some viral proteins are JDPs, and one of the best studied viral JDPs is the SV40 large T-antigen. The SV40 virus induces tumors in rodents, and large T-antigen is necessary for tumorigenesis and for diverting the host chaperone network to support virion biogenesis (112, 113). Large T-antigen is a JDP encoded in the genomes of all six polyomaviruses, which are nonenveloped viruses with double-stranded DNA structure. SV40 large T-antigen is expressed early after infection of the host cell and is essential for both viral DNA replication and transcriptional regulation. It forms hexamers and dodecamers that act as a helicase to unwind host DNA during replication and target Rb (retinoblastoma-binding protein) and p53 cellular regulatory complexes (114). Interestingly, reengineering the SV40 large T-antigen by substituting the original viral J domain with a homologous J domain from either bacteria or yeast caused a loss of the virus-specific activities of SV40 large T-antigen, emphasizing the structural importance of the SV40 large T-antigen J domain (115). Other viruses, such as the mosquito-born dengue virus, require host JDPs to replicate (116) and we are in the early days of understanding how different viruses interact with host chaperone networks.

## Inhibitors of JDP export

Compounds and small molecules that target JDP export pathways, extracellular JDPs, or JDP–substrate interactions represent a promising area of investigation, given the direct implications of JDPs in human health and the experimental tools that have emerged thus far from such studies. Export of DnaJB1 is reduced by the sphingomyelinase inhibitor, GW4869 (74) and secretion of DnaJB11/ERdj3 is inhibited by the ER/Golgi inhibitor, brefeldin A (73). DnaJC5/CSPα-mediated export of tau and α-synuclein is reduced by YM-01, a small-molecule inhibitor of Hsp70 (100) and export of aggregated Huntingtin can be reduced by the polyphenol, resveratrol. Despite an observed reduction in the export of misfolded Huntingtin, DnaJC5/CSPα release was not reduced in the presence of resveratrol, indicating this compound alters EV cargo loading rather than the export mechanism (72). JDPs themselves may also have inhibitory actions, as increased levels of the JDP, DnaJC7 was found to reduce DnaJC5/CSPα-mediated secretion (100). Finally, in a library screen of small molecules, MAL2-11B was identified as an inhibitor of large T-antigen-dependent activation of Hsp70, DNA synthesis, and viral replication (117).

## Outlook

Recent studies have revised our understanding of JDPs as more than simply intracellular chaperones and raise a number of questions about the contributions that JDPs make to the growing field of extracellular chaperone networks. JDPs are exported into the extracellular milieu as free JDPs to enhance extracellular proteostasis or as cargo within EVs to enhance proteostasis in recipient cells. Some JDPs confer resistance to chemotherapeutic agents, and other JDPs are exported in complex with aggregation-prone proteins. Yet many questions remain. Do JDPs target the same client proteins intracellularly and extracellularly? What level of JDP export is necessary to increase proteostasis capacity? When is the secretion of JDPs in complex with misfolded proteins protective and when is a mechanism of propagation and spread of disease causing proteins? There is much more to be done, and future work will provide answers to these and many more key questions.

## Conflict of interest

The author declares that there are no conflicts of interest with the contents of this article.
